# Development, validity and reproducibility of a whole grain food frequency questionnaire in Malaysian children

**DOI:** 10.1186/s12937-020-00588-y

**Published:** 2020-07-16

**Authors:** H. C. Koo, G. P. Lim, Satvinder Kaur, K. Q. Chan, Y. X. Florence Tan, X. J. Pang, L. Y. Tang

**Affiliations:** 1 Department of Bioscience, Faculty of Applied Sciences, Tunku Abdul Rahman University College, Kuala Lumpur, Malaysia; 2 Faculty of Applied Sciences, UCSI University, Kuala Lumpur, Malaysia

**Keywords:** Food frequency questionnaire, Malaysian, Children, Validation, Whole grain

## Abstract

**Background:**

To date, there is no validated whole grain assessment tool for children in any Southeast Asian countries. Hence, there is a need for a valid tool to assess whole grain intake among Malaysian children. This study aimed to develop, validate and test the reproducibility of a food frequency questionnaire (FFQ) in estimating whole grain intake among Malaysian children.

**Methods:**

A total of 392 children participated in the FFQ development and 112 children aged 9–12 years participated in the validation phase; with a subsample of 50 children participating in the reproducibility phase. Three-day diet record (3DR) as the reference method in validation phase. Spearman correlations, mean difference, Bland-Altman plot and cross-classification analyses were used to assess validity. The reproducibility was tested through a repeat administration of the FFQ, with 1 month time interval. Reproducibility analyses involved intra-class correlation coefficient (ICC), Cronbach’s alpha and cross-classification analyses.

**Results:**

The FFQ consisted of 156 whole grain food items from six food groups. Mean intake of whole grain in FFQ1 and 3DR were correlated well (*r* = 0.732), demonstrated good acceptance of the FFQ. Bland Altman plots showed relatively good agreement for both the dietary methods. Cross-classification of whole grain intake between the two methods showed that < 9.9% of children were grossly misclassified. Outcomes from ICC (0.989) and Cronbach’s alpha (0.995) demonstrated excellent reliability. All the children were classified in the same or adjacent quartile of whole grain intake.

**Conclusions:**

Overall, the findings support the validity of the developed FFQ to appropriately estimate the whole grain intake in Malaysian children. This validated FFQ will be a valuable tool for future studies, to analyses the impact of whole grain consumption with disease relationship among Malaysian schoolchildren.

## Introduction

Whole grains consist of all principal components of the grain kernel, including germ, bran and starchy endosperm [1]. The major cereal grains include rice, wheat and maize; with oats, barley, millet, rye and sorghum as minor grains [2]. In Malaysia, the most commonly consumed grains are wheat, oats, maize and rice, with wheat constituting 77.7% of the total daily whole grain intake [3]. Wheat is one of the few grains that contain vitamin A, which generating carotenoids in any useful quantity [2]. Maize, same as wheat; showed significant differences in terms of the concentration of various carotenoids [2]. Whole grain encompasses a wide selection of functional components including long-recognized traditional nutrient components and some newly-identified phytochemicals. These components suggest numerous possible mechanisms in which whole grain could exhibit health benefits of lowering risk of chronic diseases [1]. The grain germ, which is removed in the refining process, is enriched with essential vitamin E; the lack of which can lead to heart disease [2]. Evidence has consistently suggested that increasing whole grains intake is associated with lower adiposity level [4], fasting insulin levels and improved folate status among children [5].

A series of observational and interventional studies have been carried out in Asian populations to assess the whole grains intake among children [3, 6, 7]. These studies estimated the whole grains intake using 3-day 24-h dietary recalls method, reported all foods and beverages consumed over three consecutive days, followed by whole grain food identification. Ever since dietary recommendations encouraging the intake of whole grains exist in some countries, e.g. The Malaysian Dietary Guidelines for Children and adolescent includes recommendations that at least half of all grains consumed should be whole grain [8]; numerous new products based on varying contents of whole grains have become available. This situation is making the accuracy of whole grain foods’ identification and whole grains content estimation potentially difficult. It is evident that dietary assessment tools are valuable instruments to investigate the level of whole grain consumption’s adherence to dietary guidelines [9]. Hence, there is a need for a new dietary assessment approach for estimating whole grains intake.

Due to the complex nature of the diet itself, dietary assessment is notoriously difficult among the children [10]. There is a wide range of methods to assess dietary intake, from practically simple techniques e.g. dietary records, 24-h dietary recalls and food frequency questionnaires (FFQs); to the more sophisticated approach e.g. measuring biomarkers of nutrient intakes in biological specimens [10]. Each type of the dietary assessment method presents distinct set of advantages and various types of practical difficulties [11]. The commonly used methods are 24-h dietary recalls and dietary records. However, financial constraints, literacy and time constrains of both methods render them unsuitable for large-scale studies [9]. FFQs, on the other hand, are relatively more economical and practical, for both respondents and researchers [11]. It is a useful tool to provide a longer period of dietary intake information, varying from weeks to years [11]; and may focus on food items rich in a specific nutrient [12].

Predefined food list in FFQs must be validated for a target population due to several factors, e.g. ethnicities, socioeconomics and cultural differences. These factors can vary substantially between and within countries [13]. Untrustworthy information provided by an invalidated FFQ may result to attenuate disease-diet relationships [14]. It is important to validate a new FFQ. Validation studies of FFQs are normally estimate the level of measurement error, by comparing FFQ data with those of reference method, e.g. dietary records [15]. Hence, a newly developed FFQ need to be validated and tested for reproducibility. To the best of our knowledge, there are no known FFQs developed and validated specifically for assessing the whole grains intake among the Malaysian children population. This could be the first developed and validated FFQ for assessing whole grains intake in Malaysian children.

## Methods

### Participants and study design

The present cross-sectional study was conducted from October 2018 to September 2019 throughout Kuala Lumpur, Malaysia. A cluster sampling was carried out in children aged 9–12 years from a random sample selection of national primary schools in Kuala Lumpur, Malaysia. There are 298 national primary schools in three zones of Kuala Lumpur, each school was assigned a consecutive number from 1 to 298, and a total of 8 numbers were randomly selected from it. All the children who fulfilled the inclusion criteria from the 8 randomly selected national primary schools were invited to take part in the present study. Children were eligible for inclusion in the study if they were: (1) healthy children from all the ethnicity groups in Malaysia, namely Malays, Chinese, Indian, Sabah and Sarawak natives; (b) were able to write, read and understand Bahasa Melayu; and (3) present in school on the day of data collection with parental consent. Children were excluded if they had serious allergies or specific dietary restrictions affecting food intake. Ethical approval was granted by the Research and Ethnics Committee of Tunku Abdul Rahman University College. Permission to carry out data collection was obtained from the Malaysian Ministry of Education and the Kuala Lumpur Federal Territory Education Department. Approval was also sought from the principals of the chosen schools and teachers who were informed about the study. Parental consent was acquired for all children before participation. Verbal assent was also obtained from the participants prior to the study. General information including sex, ethnicity and educational year were obtained during data collection.

### Food frequency questionnaire development

FFQ was designed to estimate Malaysian children’s whole grain intake. The food list for the FFQ was developed based on a face-to-face 3-day-24-h diet recall interview, including two weekdays and one weekend day. Using Krejcie & Morgan’s formulation [16], the estimated sample was based on a total of 43,896 schoolchildren aged 9–12 years in Kuala Lumpur, a 95% confidence level, a relative precision of 5% and predicted prevalence of 50%. The sample size required for development phase was 381. Taking into account a non-response rate of 10%, the required sample size was increased to 419. Ultimately, data from 392 children were analyzed in development phase. A compendium of food pictures, food photo album [17] and a set of standard household measures such as spoons, bowls and cups of various sizes were used to aid the interview. A detailed explanation of all foods and drinks consumed by the children, including brand of products, method of cooking and mealtime were recorded. Whole grain food items derived from the diet recalls were compiled into a food list. Several whole grain food items that were thought to represent the population of interest were also included. Content validity were confirmed by three nutritionists who were familiar with whole grain foods. A total of 202 whole grain food items were organized into six main food groups: (1) bread, cake and muffin; (2) biscuit, cookie and chip; (3) ready-to-eat cereal, muesli and snack bar; (4) hot cereal drink; (5) rice, noodle and pasta; and (6) other whole grain products. Whole grain food items with similar nutritional and whole grain content were grouped together as one food item, resulting in a final whole grain food items of 156. Individual whole grain food items that could not be incorporated into any food groups were grouped in “other whole grain products”. Each whole grain food item in the FFQ was assigned a portion according to the Atlas of Food Exchanges and Portion Sizes book [17] or product packaging labels. Frequency of intake was evaluated based on a weekly basis. Frequency of consumption of each item was evaluated using nine categories: (1) never, (2) < 1 time per week; (2) 1–2 times per week; (3) 3–4 times per week; (4) 5–6 times per week; (5) once daily; (6) 2–3 times per day; (7) 4–6 times per day; and (8) > 6 times per day.

### Food frequency questionnaire validation and reproducibility

Two FFQs were collected over the course of 1 month to test the reproducibility. This short interval helped to ensure continued participation and reduce the dropout rate. The FFQs were completed through face-to-face interview. According to Willet [15], a minimum of 110 children is needed to complete FFQ1 and 3DR in a validation study. We achieved our goal by recruiting 112 children for validation phase. Whereas, a minimum sample size of 30 has been recommended for a reliability study [18]. We have successfully recruited 50 sub-sample for the reproducibility phase. Food pictures, food photo album [17] and a set of standard household measures e.g. bowls, cups and spoons of various sizes were used to aid the interview. Children were asked to indicate, on average, the portion size, frequency and number of portions of each whole grain food items (out of 156) they consumed during the past 1 week. In overall, completion of a FFQ required 20 min. None of the children had participated in both development and validation phases.

**Fig. 1:**
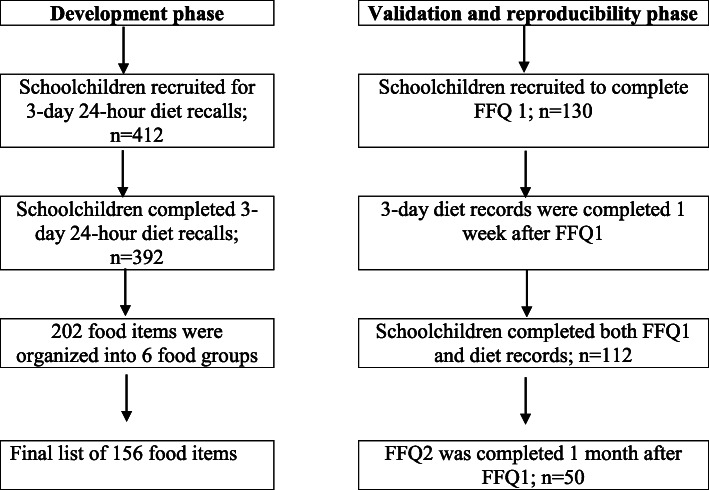
Flow chart to depict the development and validation phase of the FFQ

The reference method used to validate the results from the FFQ consisted of 3-day dietary record (3DR). Each child was asked to record 3 days food records within a week. To capture seasonal variations and weekly variations, children were asked to keep non-consecutive 3 days food records, including 1 weekend day and two weekdays. Children were asked to record the amounts of foods consumed with multiple of household tableware in order to increase the accuracy of portion size. A written instructions and food photos of various portions sizes of commonly consumed food and beverages were developed by a researcher, to assist the children in completing the 3DR form. Children were told to follow their usual diet. The 3DR forms were collected one week after the completion of the FFQ1. All 3DR were checked for completeness and children were asked for further information, where necessary. The flow chart to depict the development and validation phase of the FFQ is demonstrated in Fig. 1.

### Whole grain calculation

Whole grain foods were defined as foods made with at least one whole grain ingredient from the following grains: oat, maize, wheat, barley and rice. All the whole grain food items identified in the present study were included in the food list and data analyses, regardless of the content of whole grain [3]. The amount of whole grain per 100 g in each whole grain food was estimated by one of the three methods: (1) using quantitative ingredient declarations on food package labels; (2) directly contacting the manufacturers to obtain the information; or (3) taking an average of the whole grain content of similar products [3]. Details of all whole grain food, including the total whole grain content per 100 g, were recorded in a spreadsheet (EXCEL, Microsoft Corp., Redmond, WA, USA). For each child, the amount of whole grain consumed was estimated by multiplying the actual weight of each whole grain food consumed by the estimated percentage of whole grain content. Mean daily intake of whole grain over the 3DR were calculated. The amount of whole grain intake was calculated from the FFQ according to the following formula: frequency of intake in a week (conversion factor) X serving size X total number of servings X whole grain content in one serving.

### Statistical analyses

SPSS version 22.0 (IBM Corp., Armonk, NY, USA) was used for data analysis. The normality of the data in the validation and reproducibility studies were tested using the Kolmogorov-Smirnov test and Shapiro-Wilk test, respectively. Data was presented in median and interquartile range due to abnormality in distribution. To determine the relative validity of the FFQ against the 3DR, Spearman’s correlation coefficient was calculated. Correlation coefficients above 0.30 were considered as acceptable in FFQ validation studies [18]. Bland-Altman plot was used to assess the agreement between the FFQ and 3DR across a range of whole grain intake. Cross-classification analysis was done by segregating whole grain intake from FFQ and 3DR into quartiles, belonging to the same quartiles, adjacent (±1) quartiles, or entirely misclassified (by ≥2 quartiles). The Cohen’s cut-offs were used to interpret the level of agreement for FFQ and 3DR assessments, based on these cut-offs: (1) *r* = ±0.5 was considered strong; (2) *r* = ±0.30 was considered moderate; and (3) *r* = ±0.10 was considered weak [18]. The reproducibility of the FFQ was assessed using interclass correlation coefficients (ICC) and Cronbach’s alpha. The following definitions were used to interpret the ICC values: (1) ICC ≤ 0.40 was considered “poor”; (2) 0.41 < ICC < 0.59 was considered “fair”; (3) 0.60 < ICC < 0.74 was considered “good”; and (4) 0.75 < ICC < 1.00 was considered “excellent” [18]. The Cohen’s cut-offs were used to interpret the level of agreement for FFQ1 and FFQ2 assessments too. A *p*-value < 0.05 was considered statistically significant.

## Results

In total, 412 children aged 9 to 12 years were invited to take part in FFQ development phase; however, only 392 children consented and provided their 3-day 24-h dietary recalls, resulting in a response rate of 95.1%. Overall, there was a preponderance towards the Malay ethnicity (*n* = 282; 71.9%) in development phase. Males (50.5%) and females (49.5%) were equally distributed. The FFQ which has been developed through the list initially consisted of 202 whole grain food items. Some of the whole grain food items consist of several types and brands e.g. ready-to-eat cereals and hot cereal drinks; these whole grain food items were grouped into a same group but considered as different items, as each type and brand contained different amount of whole grain. In addition, whole grain foods that featured prominently in the diet of children population that were missed through the recall were also included into the list e.g. corn and popcorn. For easier administration, items within each whole grain food group were further grouped together based on similar nutritional content. The development of the FFQ resulted in the identification and classification of a total of 156 whole grain food items into six main food groups. Table 1 shows the food groups contribution to whole grain FFQ. There are six main groups, namely: (1) bread, cake and muffin (*n* = 7; 4.5%), e.g. whole grain bread, whole wheat cake and whole wheat paratha; (2) biscuit, cookie and chip (*n* = 33; 21.1%), e.g. Jacob’s Weetameal, McVitie’s digestive biscuit and Tesco Oaties; (3) ready-to-eat cereal, muesli and snack bar (*n* = 87, 55.8%), e.g. Yogood Crunchy muesli, Weetabix Oatibix flakes and Kellogg’s Mueslix; (4) hot cereal drink (*n* = 19, 12.2%), e.g. Ecobrown’s whole grain rice drink, Nestle Nestum with brown rice and Quaker oat cereal drink; (5) rice, noodle and pasta (*n* = 5, 3.2%). e.g. brown rice, brown rice noodle and brown rice porridge; and (6) other whole grain products (*n* = 5; 3.2%), e.g. barley, corn and quinoa. Ready-to-eat cereal, muesli and snack bar were the main whole grain food contributors (Table 1).

**Table 1:** Food groups contribution to whole grain FFQ (*n* = 156)

Food groups	Total items	Percentage of contribution (%)	Examples
Bread, cake and muffin	7	4.5	Whole grain bread, whole meal paratha, whole wheat cake
Biscuit, cookie and chip	33	21.1	Jacob’s Weetameal, McVitie’s digestive biscuit, Tesco Oaties
Ready-to-eat cereal, muesli and snack bar	87	55.8	Yogood Crunchy muesli, Weetabix Oatibix flakes, Kellogg’s Mueslix
Hot cereal drink	19	12.2	Ecobrown’s whole grain rice drink, Nestle Nestum with brown rice, Quaker oat cereal drink
Rice, noodle and pasta	5	3.2	Brown rice, brown rice noodle, brown rice porridge
Other whole grain products	5	3.2	Barley, corn, quinoa

Each type and brand of whole grain food item contained different amount of whole grain

**Fig. 2:**
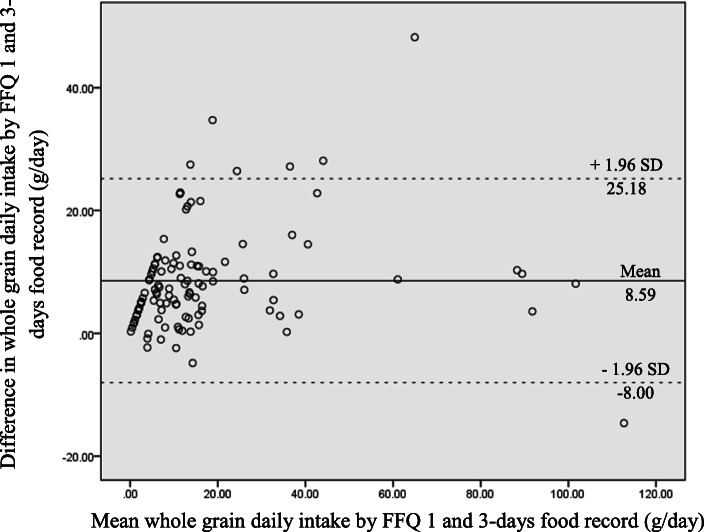
Bland-Altman plot shows agreement between the first administration of the food frequency questionnaire (FFQ1) and three-day dietary record (3DR) for whole grain daily intake. The solid line represents the mean difference in absolute intake between the two methods and the dashed line represents the limits of agreement (± 2 standard deviation [SD])

Of the 130 children recruited for FFQ validation phase, only 112 (86.2%) children successfully completed both the FFQ1 and 3DR, resulting in a response rate of 86.2%. Children in validation and reproducibility phase consisted of 49 males and 63 females, mostly from Year 4 classes (51.4%) and were of the Malay ethnicity (71.4%). Table 2 shows a comparison of the whole grain daily intake in the FFQ1 and 3DR and the correlations between the two methods. The FFQ demonstrated a significantly higher intake of whole grain (relative difference of 56.7%) than the 3DR. Overall, a strong and significant correlation between the FFQ1 and the 3DR methods (*r* = 0.732; *p* < 0.001) was obtained from the present study. The intake of whole grain from both methods was distributed into quartiles of intake and cross classified. Classification into quartiles of intake and assessment of this agreement by Cohen’s Kappa statistic is displayed in Table 3. Children were categorized as grossly misclassified if they were ranked at the extremes. Over 90.1% of children’s whole grain intake derived from FFQ and 3DR were classified into the same quartile or adjacent (±1) quartiles. Weighted kappa values was 0.429, indicating moderate agreement for both groups. The Bland-Altman plot generated shows the level of agreement between the FFQ and 3DR (Fig. 2). Its shows that the FFQ overestimated whole grain intake by an average of 8.59 g/day. There was, however, a slight proportional bias for greater differences between FFQ and 3DR with increasing whole grain intake. The limit of agreement was wide given the large standard deviation of the difference. The level of agreement between both methods was deemed to be acceptable as the scatter plot was predominantly distributed within the 95% limits of agreement.

**Table 2:** Comparison of medians and correlations for whole grain daily intake in the first administration of the food frequency questionnaire (FFQ1) and three-day dietary record (3DR) (*n* = 112)

	FFQ1	3DR	MD^a^	% MD^b^	Spearman’s correlation
	Median ± IqR	Median ± IqR			
Whole grain daily intake (g)	15.0 ± 14.5	6.5 ± 14.0	8.5	56.7	0.732^**^

^a^Median difference = median food frequency questionnaire – median food record

^b^Percentage of median difference was computed using the formula: (median FFQ1 - median 3DR) / median FFQ × 100

^**^p < 0.01

*MD* median difference, *IqR* interquartile range

**Table 3:** Cross-classification for the comparison between first administration of the food frequency questionnaire (FFQ1) and three-day dietary record (3DR) (*n* = 112)

	Cross-classification into quartiles; n (%)			
	Classified into same quartile^a^	Classified adjacently^a^	Grossly misclassified^b^	Cohen’s Kappa
Whole grain daily intake	64 (57.1)	37 (33.0)	11 (9.9)	0.429 ^***^

^a^Classified into the same or adjacent (±1) quartile

^b^Classified into opposing g quartiles (by ≥2 quartiles)

^***^p < 0.001

Difference in whole grain daily intake by FFQ 1 and 3-days food record (g/day)

One month after the FFQ1, only 50 subsample agreed to take part in reproducibility phase on a voluntary basis, and re-interviewed for FFQ2. Reproducibility was tested using intra-class correlation coefficient (ICC), Cronbach’s alpha and cross-classification between FFQ1 and FFQ2 (Table 4). The coefficient and Cronbach’s alpha for whole grain intake were 0.989 and 0.995, respectively; indicating excellent reliability. Notably, all the children (*n* = 50) were classified into the same or adjacent quartile. Weight kappa value was 0.653, indicating strong agreement for FFQ1 and FFQ2.

**Table 4:** Food frequency questionnaires at visit 1 and visit 2 – comparison of whole grain daily intake, intra-class correlation coefficient, spearman’s correlation coefficient and Cronbach’s alpha (*n* = 50)

	Median (IqR)				Cross-classification into quartiles; n (%)		
	FFQ1	FFQ2	ICC	Cronbach’s alpha	Correctly classified^a^	Grossly misclassified^b^	Cohen’s Kappa
Whole grain (g)	16.2 (15.6)	20.8 (16.6)	0.989	0.995	50 (100)	0	0.653^***^

^a^Classified into the same or adjacent (±1) quartile;

^b^Classified into opposing quartiles (by ≥2 quartiles)

^***^p < 0.001

*IqR* interquartile range, *FFQ1* first administration of the food frequency questionnaire, *FFQ2* second administration of the food frequency questionnaire, *ICC* intra-class correlation coefficient

## Discussion

FFQs are relatively more economical and practical for estimating dietary intake in epidemiology studies, as compared with other approaches [11]. FFQs may also pay particular attention on the intake of a specific nutrient [12]. Although one FFQ has been developed and validated for estimating whole grain cereal food intake among Swiss adults [19]; it might not apply correctly in Malaysian schoolchildren; due to the fact that cultural, food accessibility, preference and socioeconomic background can show a discrepancy between settings [10]. To the best of our knowledge, this is the first dietary assessment tool to specifically estimate the intake of whole grain in Malaysian children. FFQ developed in the present study was carefully designed to merge whole grain food items reflecting the dietary intake of Malaysian children population, regardless of the content of whole grain; and included whole grain items which represent a Westernized dietary pattern due to the nutritional transition and colonial influence in Malaysia [20]. It should be noted that the selection of 156 whole grain food items in development phase (Table 1), were based on previously tested methodologies [15], with emphasis on whole grain foods in the case of the 24-h diet recalls. In our FFQ, we categorized the frequency intake to minimize the loss of information [21].

Children aged 9–12 years were included in the FFQ developmental process in order to compile a list of the 156 most relevant whole grain food items from the diets of Malaysian children. According to Collin and colleagues [22], FFQs are practical and suitable dietary assessment tools to estimate dietary intake in children older than 8 years old. This age group of children are able to report their dietary intake more precisely by using FFQs, as compared with their younger counterparts [23]. However, measuring children’s dietary intake is notoriously difficult due to the portion size estimation [22]. Food pictures or food models were found to be a useful aid for the estimation of food portion sizes in children [24]. Thus, the present study developed and validated the FFQ with guided food pictures and food photo album [17] to facilitate portion sizes estimation. It is considered a useful and appropriate tool in studies of children aged 9 years and above [24].

In our validation phase (Table 2), the median of 3DR was used for evaluating the relative validity of the FFQ as the reference tool. Preferably, the use of a biomarkers e.g. plasma alkylresorcinols, should be adopted in validation studies. They are favorable dietary assessment tools to minimize the misclassification in dietary-related research [10]. However, biochemical measures are costly [10]. Thus, researchers tend to believe robust dietary assessment methods within the validation of nutritional tools. There is no agreement regarding the best method of assessing dietary intake in children [22]. Food records and 24-h diet recalls are considered acceptable methods to measure children’s dietary intake. However, food records are considered a better reference tool for validating FFQs. They are beneficial in its ability to capture all food intakes, and minimize the dependent on the children’s memory [15].

We found good correlation coefficients between FFQ and 3DR for whole grain intake in validation phase, which has been shown in Table 2. This finding is in accordance with a study, which demonstrated that interviewer-administered usually had higher correlation coefficients [25]. The wide-ranging whole grain foods items in our FFQ and its adaptation to Malaysian children dietary behaviors contributed to its good correlation coefficient too [25]. However, the performance of a FFQ should not be determined by correlation coefficients alone [26]. The percentage of median difference in whole grain intake between the two methods were greater than 10%, indicating a moderate agreement between the methods [26]. It is in consistent with Fatihah’s FFQ validation for estimating dietary intake among multiethnic Malaysian children aged 7–12 years [21]. Our results showed an overestimation of whole grain intake by the FFQ as compared to the 3DR. The wide-ranging whole grain foods items in our FFQ could potentially cause such overestimation [21]. A FFQs review, which included a total of 223 validated FFQs in their report, concluded that the FFQs consist of a median number of 79 food items, with a range of 5–350 food items [27]. On the other hand, our FFQ consists of 156 whole grain food items, which we consider to be an ideal amount of items. It can provide a more precise and accurate picture of whole grain intake in Malaysian children. Since the Malaysian Dietary Guideline for Children and Adolescent promotes the importance of whole grains [8], numerous products/ brands based on different quantities of whole grains have become available in the Malaysian market; hence, our FFQ required inclusion of various types/ brands of whole grain food items in the list, which were important to estimate the whole grain intake accurately.

FFQ systematically overestimated whole grain intake, was confirmed by the Bland-Altman plot too (Fig. 2). Bland-Altman plot indicates a case of proportional error (strong positive correlation) and systematic bias (overestimation) for whole grain intake between FFQ and 3DR. Correlation coefficient quantify the degree to which two variables are related does not necessarily imply good agreement between two methods, it is not uncommon to demonstrate acceptable correlation in the presence of bias [28]. The finding is consistent with a review on dietary assessment, which demonstrated that FFQs have potential in overestimating food consumption; on the other hand, diet records tend to underestimate dietary intake [29]. The agreement between the FFQ and 3DR was tested by cross-classification analysis too. Classification whole grain intake among children was performed in this analysis [21]. Our FFQ demonstrated a small degree of misclassification. Fatihah and colleagues [21] demonstrated the similar outcome in her FFQ validation to estimate dietary intake in Malaysian children.

FFQ was administered twice to test the reproducibility. Two FFQs were collected over the course of 1 month. The respective time interval was set to minimize any potential progressive changes in whole grain consumption among children [10]. In reproducibility phase, ICC was applied to test the reproducibility (Table 4). Intra-class correlation investigates the consistency; whereas, coefficient demonstrates the degree of agreement between FFQ1 and FFQ2 [18]. Our FFQ obtained excellent reproducibility outcomes from ICC. The outcome is unsurprising given than repeat administrations of the FFQ2 were only 1 month apart from FFQ1 [25]. A Cronbach’s alpha more than 0.70 indicates the FFQ is reliable [30]. Cronbach’s alpha in the present study demonstrated excellent agreement between FFQ1 and FFQ2. Further, using quartile classification, we found that all the children were classified into the same or adjacent quartile, and the weighted kappa value indicated strong agreement for FFQ1 and FFQ2. These results are comparable to Lebanese [10] and Malaysian [21] studies performed in children.

Although the outcomes are promising, it has a number of limitations that need to be considered. It is well-recognized that 3DR is not the best dietary assessment tool, but there is no agreement regarding the gold standard for validating FFQs [27]. The 3DR is helpful in its ability to capture all food intakes. A validated FFQ to assess general dietary intake in Malaysian children has shown the high level of accuracy by using 3DR as reference method [21]. Further, a review which compared between self-reported dietary assessments and biomarkers demonstrated that, the correlations between both methods were similar using a 3DR [31]. Thus, we collected 3DR to minimize the bias. Another limitation was that, the social desirability bias in self-reported dietary assessment may compromise the validity of dietary intake masures [27]. In order to minimize such a bias, researchers were trained to limit any judgmental during the completion of the dietary assessments. Besides, researcher had re-assured the children of their anonymity and confidentially of individual results. Another limitation that ought to be considered was the whole grain content analyses. In the context of the study, there exists no whole grain content analyses database in Malaysia. Hence, we adopted the methods from a whole grain national study in Malaysia [3]. The use of those methods could have led to miscalculation of whole grain intake estimations, as children may not able to recognize correctly all the whole grain foods; however, it is less likely to have affected the validity and reliability measures of the developed FFQ, since these methods were used in the analyses of whole grain intake of both the FFQ, as well as the 24-h diet recalls and 3DR.

Despite these limitations, the present study has a number of strengths worth noting. To the best of our knowledge, this is the first developed whole grain FFQ specifically for Malaysian children; and the robust steps taken to ensure the validity and reproducibility of the FFQ [27] represent the main strengths of our study. Although one FFQ related to the whole grain intake and adapted to Swiss adults has been published [19], but it might not be suitable for Malaysian children; due to the fact that cultural and preference can show a discrepancy between settings [10]. While FFQs validated for Malaysian children populations were mostly developed to assess general dietary intake [21, 32], our FFQ was developed specifically to estimate whole grain intake. Our interview-administered FFQ has potential to minimize any possible bias and mistake, by providing immediate feedback to the children [10].

## Conclusion

To the best of our knowledge, this is the first newly developed and validated FFQ for estimating whole grain intake in a sample of Malaysian children. The developed FFQ may be used in population-based studies aiming at monitoring whole grain intake and whole grain food consumption patterns amongst Malaysian children. This validated FFQ will be a valuable tool for future studies analyzing the impact of whole grain consumption with disease relationship within this age group.

## Data Availability

The food frequency questionnaire used and/ or analyzed during the current study are available from the corresponding author on reasonable request.

## Abbreviations

24HR: 24-h diet recall
3DR: 3-day diet record
FFQ1: First administration of FFQ
IqR: Interquartile range
ICC: Intra-class correlationcoefficient
FFQ2: Second administration of FFQ
